# Early detection of *Dendrolimus* species infestations: integrating UAV hyperspectral and LiDAR data

**DOI:** 10.3389/fpls.2025.1664466

**Published:** 2025-11-06

**Authors:** Rui Tang, Linfeng Yu, Peiyun Bi, Quan Zhou, Xudong Zhang, Lili Ren, Youqing Luo

**Affiliations:** 1 Beijing Key Laboratory for Forest Pest Control, Beijing Forestry University, Beijing, China; 2 Institute of Forest Resource Information Techniques, Chinese Academy of Forestry, Beijing, China

**Keywords:** *Dendrolimus* species, hyperspectral, lidar, early monitoring, canopy structure, UAV remote sensing

## Abstract

*Dendrolimus* species are the major defoliating forest pests in China, causing severe damage to pine forests. Establishing an effective early monitoring system was crucial for timely implementation of control measures to prevent further infestation, significantly reducing economic losses and ecological damage. While previous studies have demonstrated the limited effectiveness of spectral data alone in early detection of *Dendrolimus* spp. infestations, our research reveals that needle loss is the primary damage symptom, whereas canopy structural characteristics remain underexplored in early monitoring. To address this knowledge gap, this study innovatively integrates unmanned aerial vehicle-based hyperspectral imaging (HSI) with Light Detection and Ranging (LiDAR) data. This study employed SPA, ISIC, and ISIC-SPA algorithms in combination with Random Forest (RF) to select sensitive hyperspectral imaging (HSI) bands. Subsequently, vegetation indices (VIs) were developed from these optimal wavelengths and integrated with LiDAR metrics. Finally, the performance of RF models trained on individual data sources (HSI VIs or LiDAR metrics) and on the combined data (HSI+LiDAR) was evaluated for detecting *Dendrolimus* spp. damage at the individual tree level. For HSI band selection, compared to the 10 bands selected by SPA-RF (OA = 71.05, Kappa=0.57) and the 21 bands selected by ISIC-RF (OA = 75.44, Kappa=0.63), ISIC-SPA-RF (OA = 70.18, Kappa=0.55) selected only 3 bands and achieved good classification results on the validation set, which substantially reduced data redundancy and improved VI construction. For individual tree-level detection of *Dendrolimus* spp. damage, four VIS and seven LiDAR-derived metrics were utilized. The results showed that the HSI method (OA = 72.81%, Kappa=0.59) outperformed the LiDAR method (OA = 71.05%, Kappa=0.56). The combined data approach achieved the highest overall accuracy (OA = 83.33%, Kappa=0.75), with an early detection accuracy of 82.93%, which was significantly better than using HSI or LiDAR data alone. Our study demonstrates that LiDAR can effectively capture the spatial distribution changes of needles caused by defoliation, while also revealing spectral reflectance characteristics in the near-infrared (NIR) band. The integration of HSI and LiDAR data significantly enhances the early detection accuracy for *Dendrolimus* spp. infestations. This approach not only provides critical technical support for *Dendrolimus* spp. control, but also establishes a novel remote sensing methodology for monitoring other defoliation pests.

## 1 Introduction

The pine caterpillars (Lepidoptera, Lasiocampidae, *Dendrolimus* species) are typical leaf-feeding pests and major forestry pests in China, causing severe damage to important tree species such as pine, cypress, and fir (Bao et al., 2024). *Dendrolimus* spp. feed on conifer needles, disrupting the ecological and structural functions of coniferous forests and causing tree mortality in severe cases (Han et al., 2024). Additionally, Damage caused by *Dendrolimus* spp. weakens tree potentials thereby increasing the risk of secondary pest infestations by wood-boring pests (Song et al., 2016). China’s large-scale plantations are dominated by monoculture coniferous forests characterized by simplified structures and low resistance (Bao et al., 2022). By 2023 the infestation area of *Dendrolimus* spp. in China reached 60.04 million hectares thereby causing significant economic and ecological losses (Liu et al., 2024). Effective management and control of *Dendrolimus* spp. are therefore essential.

In Liaoning Province, the major *Dendrolimus* spp. pest species include *Dendrolimus tabulaeformis* Tsai et Liu, *Dendrolimus superans* Butler, and *Dendrolimus spectabilis* Butler. *Dendrolimus* spp. have one generation per year. Newly hatched larvae feed on one side of pine needles, creating characteristic notches. These larvae are unable to fully consume individual needles. Damaged needles exhibit yellowing, desiccation, and curling. *Dendrolimus* spp. overwinter as larvae in the soil or under bark. In the following year, overwintering larvae ascend the tree, disperse across branches, and rapidly consume entire needles, thereby entering their peak feeding phase. Following emergence, adults disperse to nearby forest stands or migrate to more distant forest stands to lay eggs. *Dendrolimus* spp. outbreaks are cyclical, cover large areas, and escalate rapidly (Zhang et al., 2022). During the early larval feeding stage, physical and chemical methods can effectively prevent further damage and spread (Skrzecz et al., 2020). However, monitoring remains a critical phase for implementing effective control strategies. Traditional ground surveys require significant labor and time, making them unsuitable for large-scale pest monitoring. They also risk missing optimal control periods (Nasi et al., 2015).

The rapid development of remote sensing technology enhances forest health monitoring capabilities and provides a critical tool for acquiring forest pest and disease data (Luo et al., 2022). Satellite-based studies on *Dendrolimus* spp. monitoring are primarily conducted at the stand level (Zhu et al., 2016; Zhao et al., 2024; Zhang et al., 2025). Although satellite monitoring demonstrates the ability to detect forest changes induced by *Dendrolimus* spp., it remains constrained by insufficient revisit frequency, potentially missing early stage infestations (Hu et al., 2024). In early stage monitoring, the high resolution of hyperspectral sensors can detect more detailed and precise spectral changes. UAV-based hyperspectral imaging (HSI) captures extensive narrow-band spectral information from horizontal tree canopies, enhancing tree health detection. This is critical for monitoring forest pests and diseases in early stage infestations or complex ecological environments (Zhang et al., 2019). Studies on wood-boring pests *Bursaphelenchus xylophilus*, *Agrilus planipennis*, and *Dendroctonus valens* used HSI and achieved good early detection accuracy (Pontius et al., 2008; Li et al., 2023; Bi et al., 2024). In studies on leaf-feeding pests, Zhang et al. developed an ISIC-SPA-P-PLSR framework using hyperspectral data to estimate defoliation percentages (DP) in trees damaged by *D. tabulaeformis* The coefficient of determination (R^2^) reached 0.8061 for trees at mild to moderate damage stages (Zhang et al., 2018). Bai et al. developed a regression model for DP based on five spectral VIS, achieving an R² of 0.786 (Bai et al., 2016). While tree damage caused by *Dendrolimus* spp. has been distinguishable using hyperspectral technology in previous studies, canopy spectral information can be lost or overall reflectance curves distorted due to shadows caused by variations in camera angles and solar elevation angles (Liu et al., 2025). Furthermore, in pine forests, healthy canopies and damaged branches are often interwoven and obscured, making such overlapping structures difficult to accurately characterize through spectral data alone (Lin et al., 2021, 2023). Therefore, precise monitoring at mild damage stages continues to be faced with significant challenges.

Throughout the entire infestation cycle, larvae of *Dendrolimus* spp. continuously feed on all needles within the host tree’s crown, inducing conspicuous morphological alterations and biomass loss – a symptomatic profile distinctly divergent from that of wood-boring pests, whose infestation typically manifests as crown discoloration. Previous studies on remote sensing monitoring of *Dendrolimus* spp. primarily relied on spectral data, which exhibited limitations in capturing dynamic variations within lower canopy layers and were additionally subject to weather-related interferences.

As an active light detection and ranging (LiDAR) also corrects reduced spectral intensity and altered spectral shapes in hyperspectral imagery caused by shaded areas, improving spectral information (Oduncu and Yuksel, 2021). Wang et al. demonstrate that combining HSI and LiDAR data enables individual tree segmentation in high-density areas (Wang et al., 2025), This approach enhances early detection at the individual tree level. Previous studies in forest pest monitoring showed that combining HSI data and LiDAR data can improve accuracy. LiDAR derived structural metrics also detected forest pests (Lin et al., 2019; Yu et al., 2021; Zhou et al., 2022). However, the combination of HSI and LiDAR data has not been used in remote sensing monitoring of *Dendrolimus* spp.

Based on the characteristic of *Dendrolimus* spp. damage leads to needle loss, this study aim to investigate the role of HSI and LiDAR data in the early monitoring of *Dendrolimus* spp. The objectives are: (1) to identify the most sensitive HSI bands and LiDAR structural indices for detecting the early stage of *Dendrolimus* spp. infestation at the individual tree level; (2) to compare the differences between HSI and LiDAR in early monitoring of *Dendrolimus* spp.; (3) to explore the potential of integrating HSI and LiDAR data for monitoring *Dendrolimus* spp. in early damage stages.

## 2 Materials and methods

### 2.1 Study area

**Figure 1:**
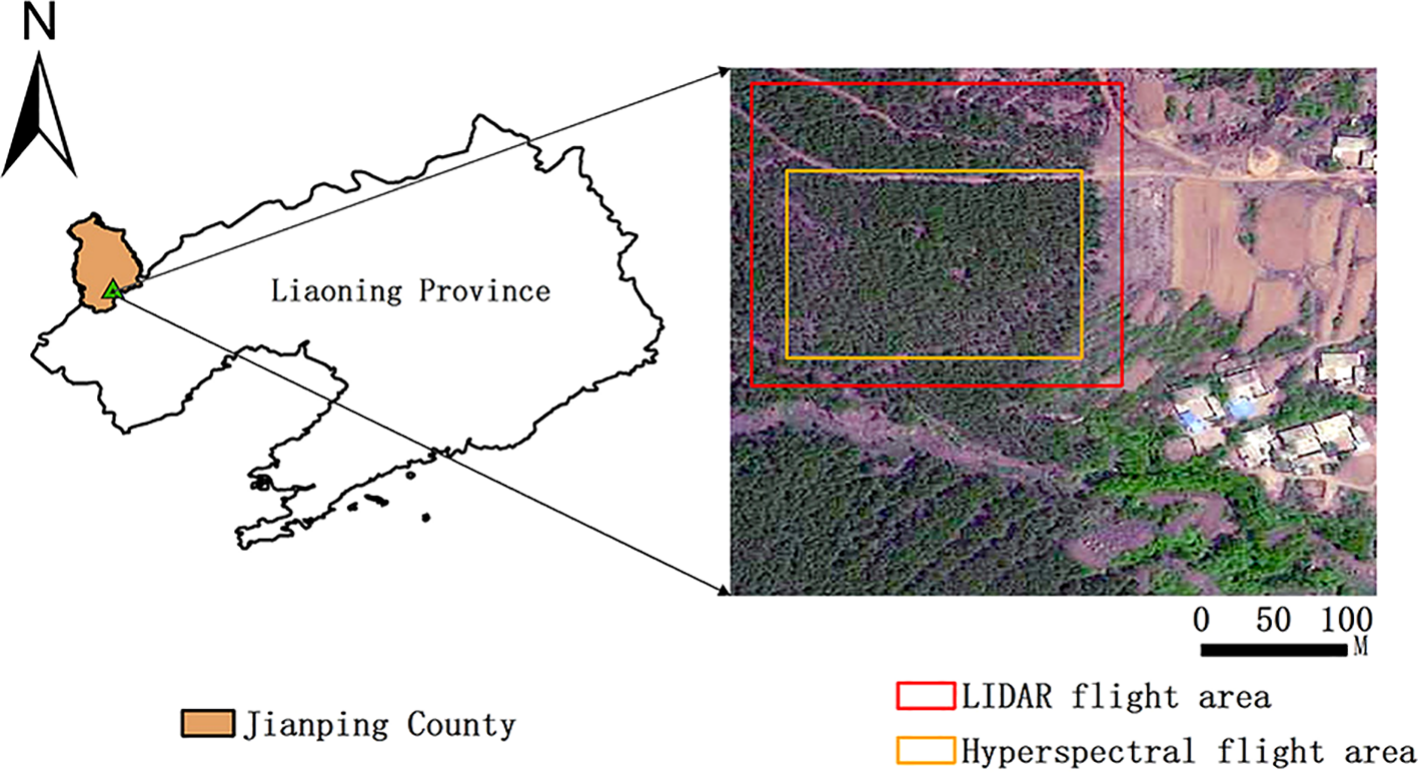
Study area.

The study area located in Yushulinzi Township, Jianping County, Chaoyang City, Liaoning Province, China. The county’s topography consists mainly of mountainous and hilly terrain. Average annual temperatures range from 5.4°C to 8.7°C, with elevations varying between 400 and 1200 meters above sea level. A semi-humid to semi-arid continental monsoon climate prevails in the region. *Pinus tabuliformis* is the dominant tree species. A pure *P. tabuliformis* stand infested with *Dendrolimus* spp. was selected as the test plot based on a ground survey (**Figure 1**).

### 2.2 Remote sensing data acquisition and processing

#### 2.2.1 Field survey

Based on field surveys and previous studies, *P. tabuliformis* growth showed no significant negative effects at DP ≤ 30%. Instead, DP ≤ 30% may promote tree height, apical shoot growth, lateral shoot growth, and improve tree vigor. Based on field surveys, the mild damage stage (DP ≤ 30%) was defined as the early stage of *Dendrolimus* spp. Infestation. However, when DP≥ 50%, tree growth decreased with increasing DP, causing significant negative impacts on height and radial growth (Zhou and Li, 1993; Yang and Li, 1994). Based on the relationship between DP and tree growth, damaged trees were divided into three stages: mild stage (DP ≤ 30%), moderate stage (30% < DP < 50%), and severe stage (DP ≥ 50%). The mild damage stage (DP ≤ 30%) was defined as the early stage of *Dendrolimus* spp. damage.

**Figure 2:**
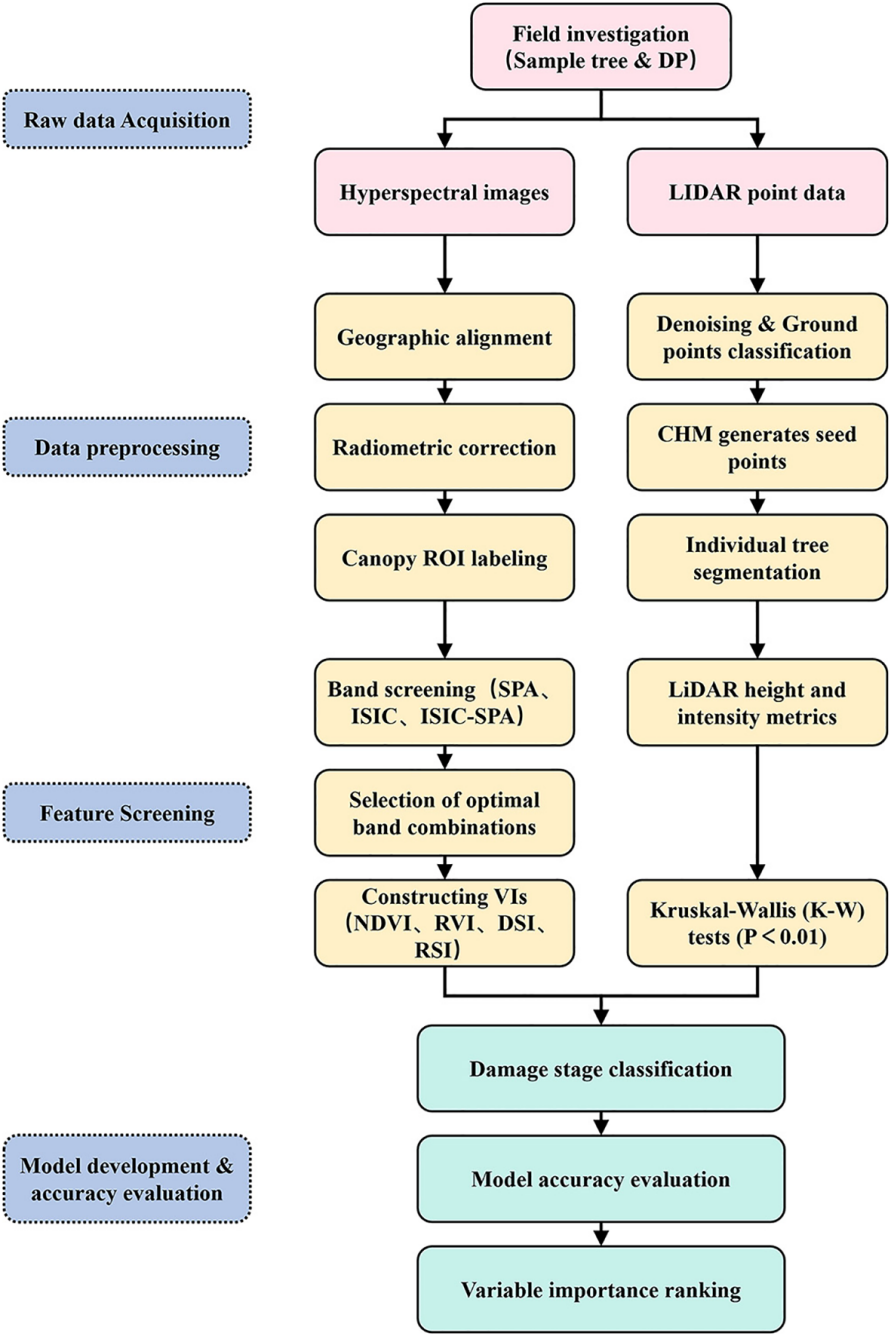
Overall experimental workflow.

**Figure 3:**
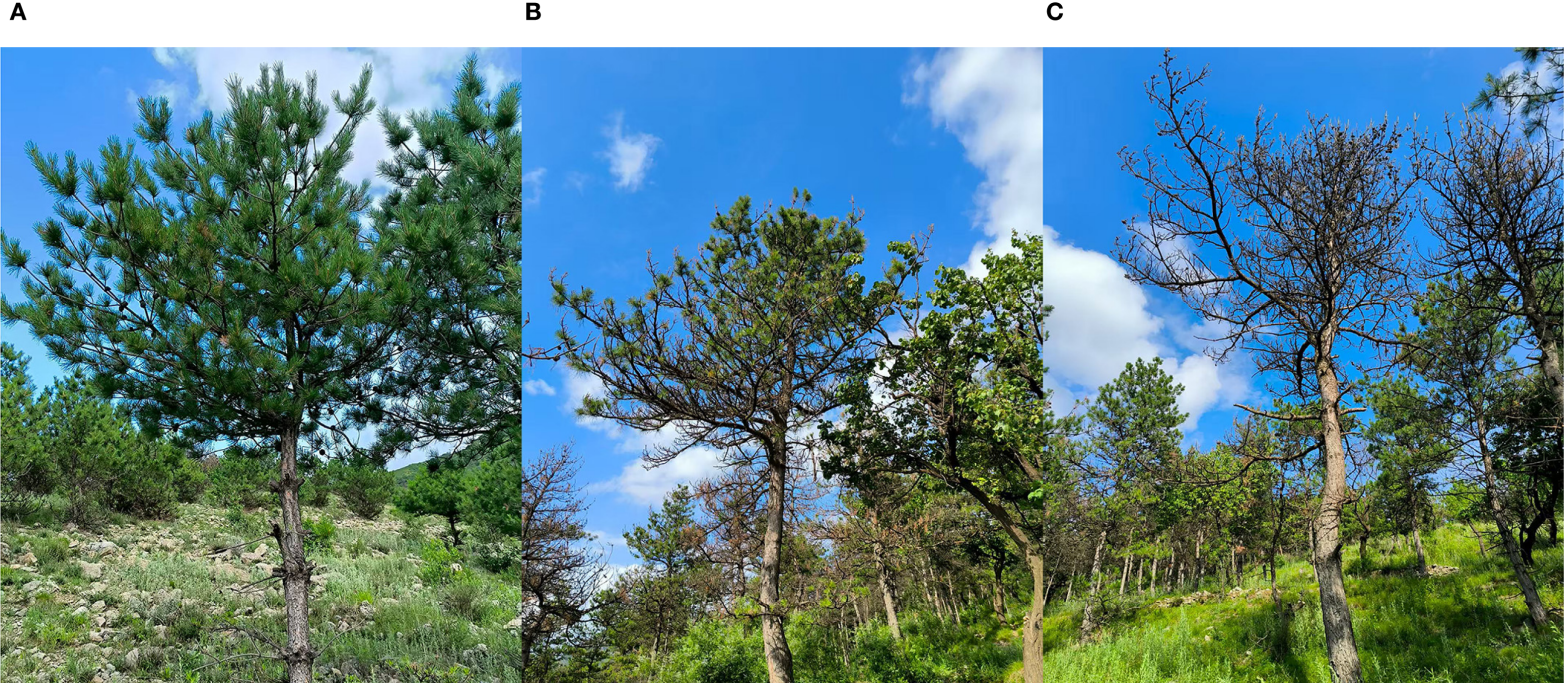
The three stages of damage in *P. tabuliformis*. **(A)** Mild damage stage; **(B)** Moderate damage stage; **(C)** Severe damage stage.

Data were collected from May 28 to June 7, 2024 (**Figure 2**). The DP was measured to quantify tree damage. Standard branches representing the overall defoliation of each sample tree were selected. Each tree was divided into three vertical layers. Within each layer, standard branches from the east, south, west, and north directions were systematically selected. The ratio of damaged needles to total needles was recorded for each standard branch. The average DP of all standard branches was calculated as the DP of the sample tree. Based on DP values, sample trees were classified into three damage stages (**Figure 3**): mild (DP ≤ 30%), moderate (30% < DP < 50%), and severe (DP ≥ 50%). A total of 284 individual sample trees were included in the ground survey. These trees were divided into 103, 99, and 82 samples with mild, moderate, and severe damage. The study employed a DJI Mavic 3M (DJI, Shenzhen, China) equipped with dual imaging systems—a 4/3-inch CMOS visible light camera and a 1/2.8-inch CMOS multispectral camera—to acquire RGB orthophotographic data and geotag sample tree locations. This configuration enabled simultaneous high-resolution visible spectrum documentation and multispectral analysis for vegetation monitoring application.

#### 2.2.2 UAV hyperspectral data acquisition and processing

**Figure 4:**
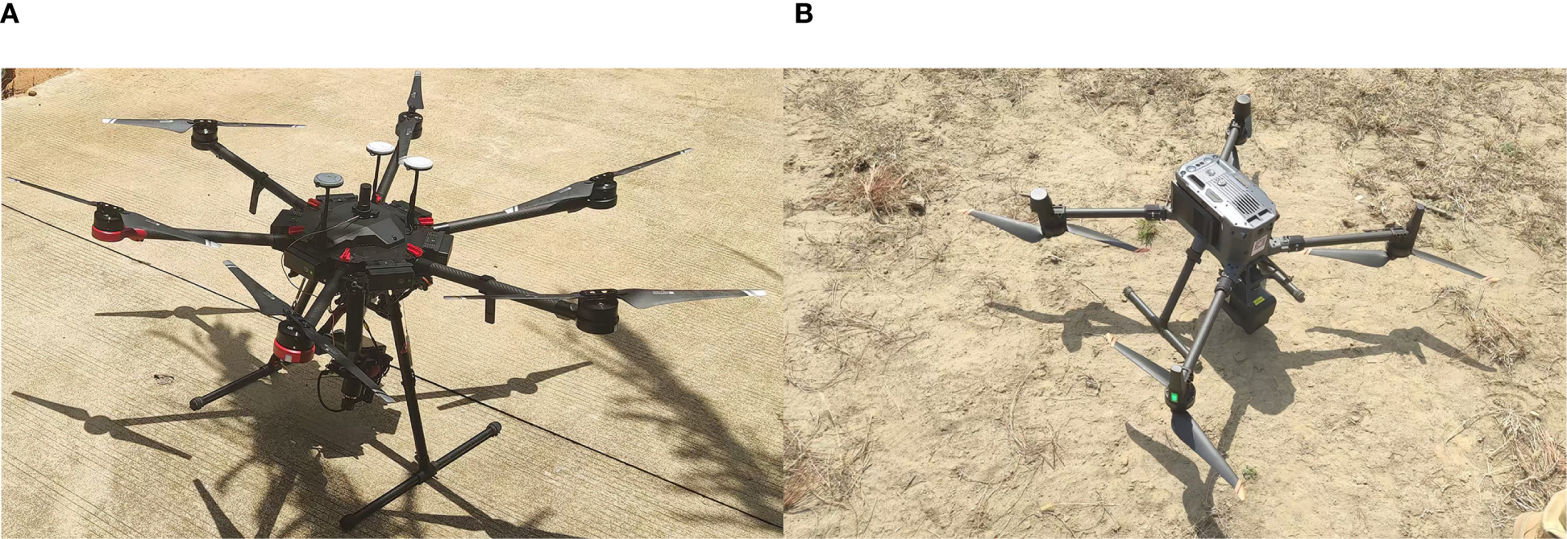
UAV hyperspectral and LiDAR systems. **(A)** DJI Matrice M600 Pro (HSI); **(B)** DJI Matrice 350 RTK (LiDAR).

Hyperspectral data were acquired on May 30, 2024 (10:30 am–12:00 pm), under clear and windless conditions. A DJI Matrice M600 Pro hexacopter UAV (DJI, Shenzhen, China, **Figure 4A**) equipped with a PikaL hyperspectral camera (Resonon, Bozeman, MT, USA) was deployed. The spectral range is 400–1000 nm, with a spectral resolution of 4 nm. The camera has a field of view (FOV) of 17.6 and a focal length of 17 mm. The flight was conducted at an altitude of 100 m with 50% heading and lateral overlap maintained. Weather conditions were clear. The measurement area was extensive. Two flight missions were conducted. A 3 m² reference cloth was placed in the flight area for radiometric calibration and reflectance correction. UAV inertial navigation and Z-survey i50 RTK enhanced POS accuracy.

Raw hyperspectral POS data were processed using SBGcenter3.4.89 software (Company, Country) with differential correction. Route segmentation boundaries were established in Omap10.0.5 (Company, Country). Original hyperspectral data were pre-segmented in AirlineDivision1.8 (Company, Country) based on route boundaries. Pre-segmented data from both missions were merged in Megacube2.9.6.3 (Company, Country). Geographic elevation was set to 650 m in ArcGIS10.7 (Company, Country). Sequential alignment of route images was performed in ArcGIS10.7 using existing RGB orthophotos. Aligned images were mosaicked in ENVI Classic5.6. A hypercube file was generated in MegaCube2.9.6.3 and converted to a reflectance image in ENVI5.6. The region of interest (ROI) tool in ENVI5.6 was used to delineate the crowns of 284 sample trees in hyperspectral imagery. The spectral reflectance of the canopy areas was calculated, and the average reflectance across 145 spectral bands was obtained.

#### 2.2.3 UAV LiDAR data acquisition and processing

On the morning of May 28, 2024 (07:00–08:40), under windless and rain-free conditions, point cloud data acquisition was conducted using a DJI Matrice 350 RTK platform (DJI, Shenzhen, China; **Figure 4B**) equipped with a Zenmuse L2 LiDAR sensor (DJI, Shenzhen, China). Flight planning utilized a DJI Pilot 2 system, with data collection parameters set to 240 kHz pulse frequency, repetitive scanning mode, and five-echo reception. The RTK state was FIX, the laser side overlap rate was 60%, the flight speed was 15m/s, the flight altitude was set to 100m, and the point cloud density was 189 points/m^2^. LiDAR data processing was conducted in DJI Terra v4.6.6, followed by exportation of LAS-format point cloud files. Noise removal and point classification were performed in LiDAR360 (GreenValley Inc., Shanghai, China) using the Improved Progressive TIN Densification (IPTD) algorithm, separating ground and non-ground points. Ground point normalization yielded three raster products: a digital elevation model (DEM), digital surface model (DSM), and canopy height model (CHM). Individual tree seed points were subsequently derived from the CHM. Over-segmented and under-segmented areas were adjusted by adding or deleting seed points using ALS seed point editing. Individual tree point clouds were segmented using seed points. Individual tree boundaries were delineated using a concave hull algorithm with an edge length of 0.2 m. A total of 5 height metrics and 5 intensity metrics were computed using a forest parameter analysis tool, with a height threshold set to 0.5 m.

### 2.3 Spectral band selection

**Figure 5:**
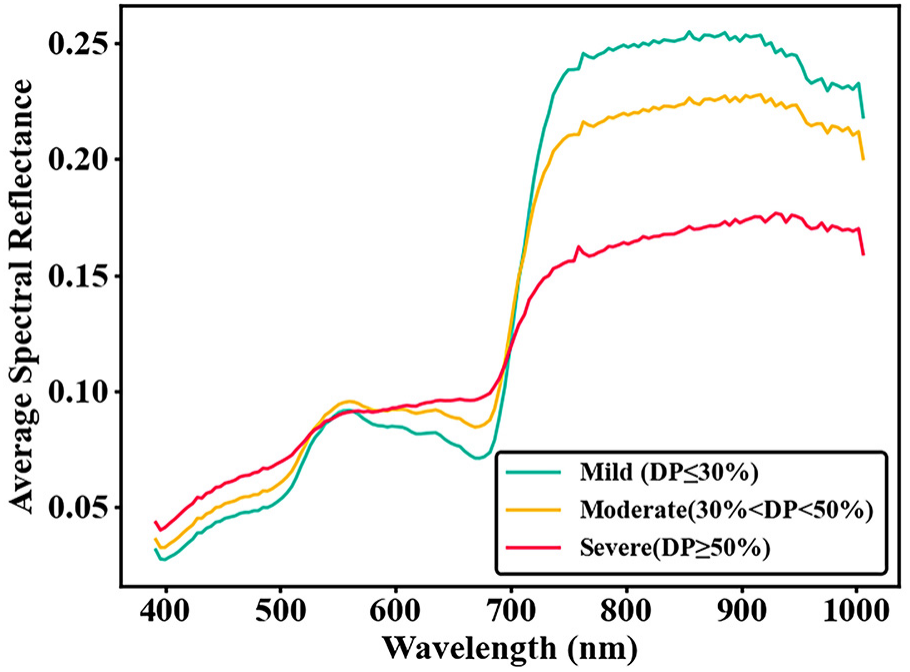
Average spectral reflectance at different damage stages.

Hyperspectral data provide rich spectral information (**Figure 5**). However, adjacent bands are often highly correlated, leading to significant redundant information and computational complexity (Lorencs et al., 2018; Han et al., 2022). Compared to feature extraction, band selection preserves the original hyperspectral features and biophysical significance (Li et al., 2022). This method eliminates redundant information and accelerates analysis speed. In this study, the Successive Projections Algorithm (SPA) (Jiang et al., 2022; Ma et al., 2022; Bai et al., 2024) and the Instability Index between Classes (ISIC) (Zhang et al., 2018, 2019; Wang et al., 2023) were applied to screen full-band data. Based on experimental results, the ISIC-SPA (Zhang et al., 2018) method was further utilized for secondary band selection.

#### 2.3.1 Three band selection algorithms

SPA is a forward iterative search algorithm designed to minimize collinearity between vectors (Sun et al., 2019). Starting with one variable, the algorithm selects the next variable in the orthogonal subspace of the previously selected variable. This new variable has the maximum projection value and the smallest collinearity (Liu et al., 2023). By choosing variables with the least redundant information, SPA effectively reduces linear relationships between variables, thereby lowering multicollinearity (Feng et al., 2022). Band combinations selected by SPA were used to construct Random Forest (RF) models. The optimal number of bands was determined based on the 10-fold cross-validated accuracy (10-CV ACC) of the training set.

The instability index between classes (ISIC) quantifies the separability of HSI bands. ISIC values determine the suitability of bands for classification tasks. Bands suitable for classification show smaller ISIC values, while unsuitable bands show larger ISIC values. Spectral bands are classified into three categories (mild, medium, severe) using ground-survey DP. For three or more categories, ISIC is calculated with its specific formula:


$$\text{ISICi}=\frac{\text{Δwithin},\text{i}}{\text{Δbetween},\text{i}}=\frac{\text{m}}{\text{m}(\text{m}-1)}\sum_{\text{z}=1}^{\text{m}-1}\sum_{\text{j}=\text{z}+1}^{\text{m}}=\frac{{\text{S}}_{\text{z},\text{i}}+{\text{S}}_{\text{j},\text{i}}}{{\text{m}}_{\text{z},\text{i}}-{\text{m}}_{\text{j},\text{i}}}$$

This study employed D_i_, defined as the absolute difference between ISIC values of neighboring bands, to evaluate the suitability of spectral bands for classification. The removal of bands with higher D_i_ values improved both classification efficiency and accuracy (Zhang et al., 2018).


$$D_{i}=|{\text{ISIC}}_{\text{i}}-{\text{ISIC}}_{\text{i}+1}|$$

The optimal threshold D_i_ was determined using ISIC combined with RF. An initial large step size was set to define the threshold range based on the 10-CV ACC of the training set at selected thresholds. Iterative reduction of the step size was applied to identify the final optimal threshold (Wang et al., 2023).

Single band selection methods risk losing critical HSI information. Redundant data may persist (Zhang et al., 2024). The ISIC-SPA method was further applied to screen bands; it eliminates redundant combinations from ISIC, enhances information independence, and provides SPA with a stable denoised band subset for classification. This reduces the number of bands processed by SPA, shortens runtime, and improves selection efficiency. The process of combining three band selection algorithms with the RF model for HSI band selection is defined as SPA-RF, ISIC-RF, and ISIC-SPA-RF. Band combinations from three methods were used to build RF models. Classification performance on the same test set was compared, and model fitting was evaluated to determine the optimal combination.

#### 2.3.2 Random forest model parameter configuration and model evaluation

RF is a classifier composed of multiple decision trees. It has advantages including insensitivity to parameters, resistance to overfitting, suitability for small sample sizes, and strong performance in group classification (Jiang et al., 2021; Bi et al., 2024). The random forest classifier was implemented through the RandomForestClassifier module in Python’s sklearn.ensemble library. Hyperparameter optimization focused on two critical variables: the quantity of decision trees (n_estimators) and the initialization seed (random_state). The dataset was split into a 6:4 ratio. 170 samples formed the training set for model training and 10-fold cross-validation, and 114 samples formed the test set for independent testing.

Classification accuracy was quantitatively evaluated using metrics derived from the confusion matrix: Overall Accuracy (OA), Kappa coefficient, Producer’s Accuracy (PA), and User’s Accuracy (UA). PA represents the ratio of correctly classified samples to the actual number of samples in a class. UA represents the ratio of correctly classified samples to the total number of samples predicted as that class by the model (Murfitt et al., 2016).

#### 2.3.3 Construction of vegetation indices

To enhance the information contained in the spectral bands and reduce the influence of factors such as soil background, 4 band combination methods were selected based on previous studies and as shown in **Table 1**. All possible combinations of these bands were generated, and Spearman correlations between each vegetation index (VI) and three damage stages of *P. tabuliformis* were analyzed. The VI with the highest correlation in each category was selected as a classification variable (Ma et al., 2021).

**Table 1:** Vegetation indices selected.

Vegetation index	Formula
NDSI	$\frac{\left({\text{R}}_{\text{x}1}-{\text{R}}_{\text{x}2}\right)}{\left({\text{R}}_{\text{x}1}+{\text{R}}_{\text{x}2}\right)}$
DSI	${\text{R}}_{\text{x}1}-{\text{R}}_{\text{x}2}$
RSI	$\frac{{\text{R}}_{\text{x}1}}{{\text{R}}_{\text{x}2}}$
RA	$\frac{{\text{R}}_{\text{x}1}}{\left({\text{R}}_{\text{x}2}+{\text{R}}_{\text{x}3}\right)}$

### 2.4 Significance analysis and variable importance ranking

Kruskal-Wallis (K-W) tests were applied to the LiDAR metrics from Section 2.2.3 and the VIs from Section 2.3.3. Variables showing significant differences across damage stages were selected as inputs in the combined dataset to construct the RF classification model. Variable importance in the RF model was assessed using the Mean Decrease Accuracy (MDA) index. The assessment involved randomly permuting the values of each variable in the out-of-bag (OOB) samples and comparing the misclassification rates before and after permutation (Archer and Kirnes, 2008; Verikas et al., 2011).

## 3 Results

### 3.1 Spectral band selection

**Figure 6:**
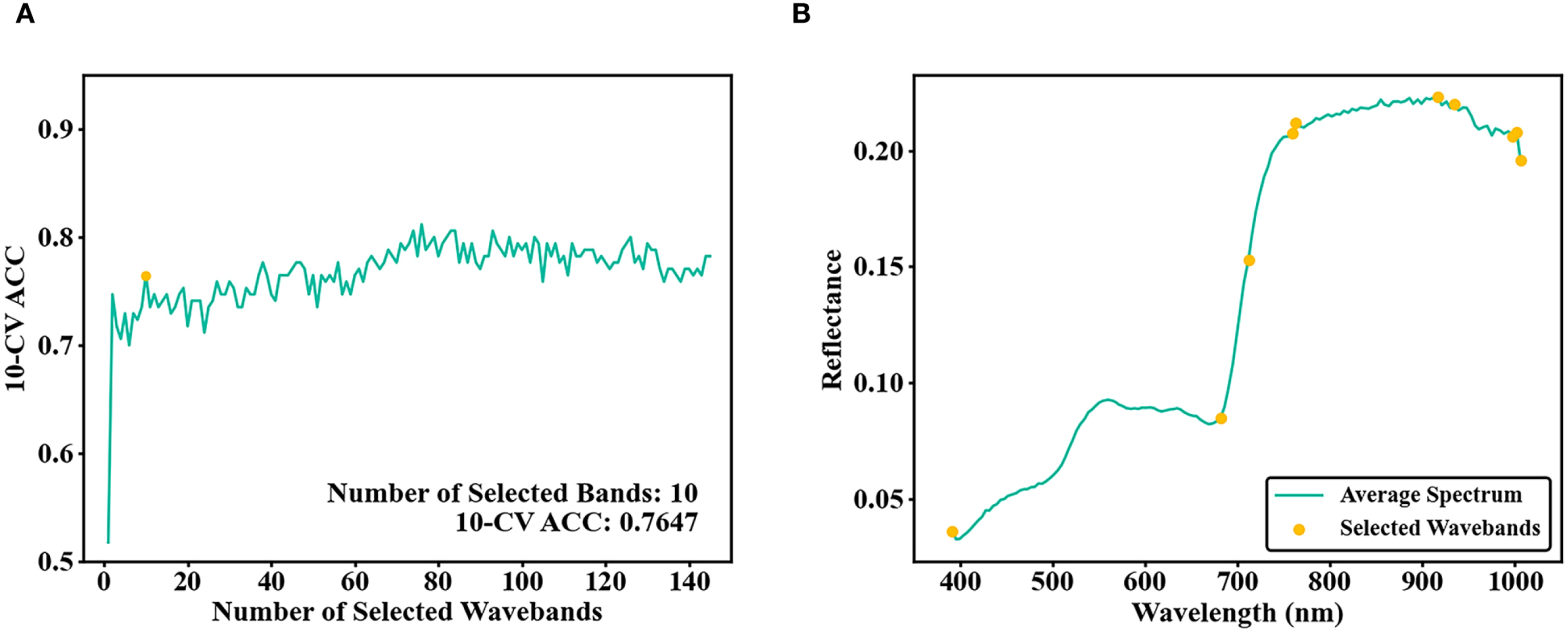
**(A)** Number of bands selected by SPA and 10-CV ACC of the training set; **(B)** SPA selected bands.

The SPA-RF results for optimal band selection were shown in **Figure 6**. As the number of selected bands increased, the 10-CV ACC of the training set showed small fluctuations. The overall trend first rose and then fell, eventually stabilizing near 0.76. Considering both fitting accuracy and band quantity, SPA-RF selected 10 bands: 5 near-infrared (NIR) bands, 4 infrared bands, and 1 ultraviolet band. At this stage, the training set achieved a 10-CV ACC of 0.7647.

**Figure 7:**
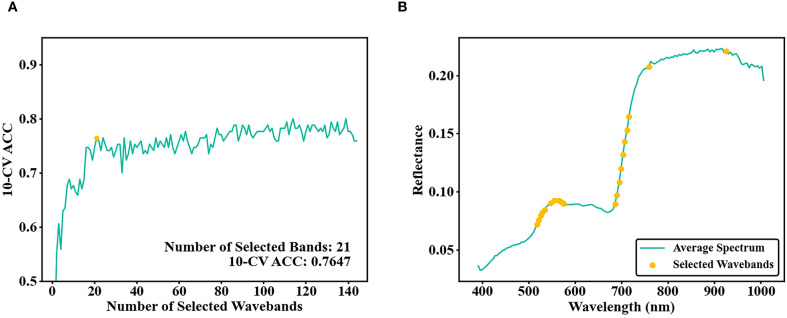
**(A)** Number of bands selected by ISIC and 10-CV ACC of the training set; **(B)** ISIC selected bands.

RF models were built with HSI bands selected based on different thresholds. Training performance improved with increasing band numbers. Beyond 21 bands, no substantial increase in training set 10-CV ACC was observed. The ISIC threshold was therefore set to 0.00637. Bands with Di values exceeding this threshold were removed. A final selection of 21 bands achieved a 10-CV ACC of 0.7647 (**Figure 7**).

**Figure 8:**
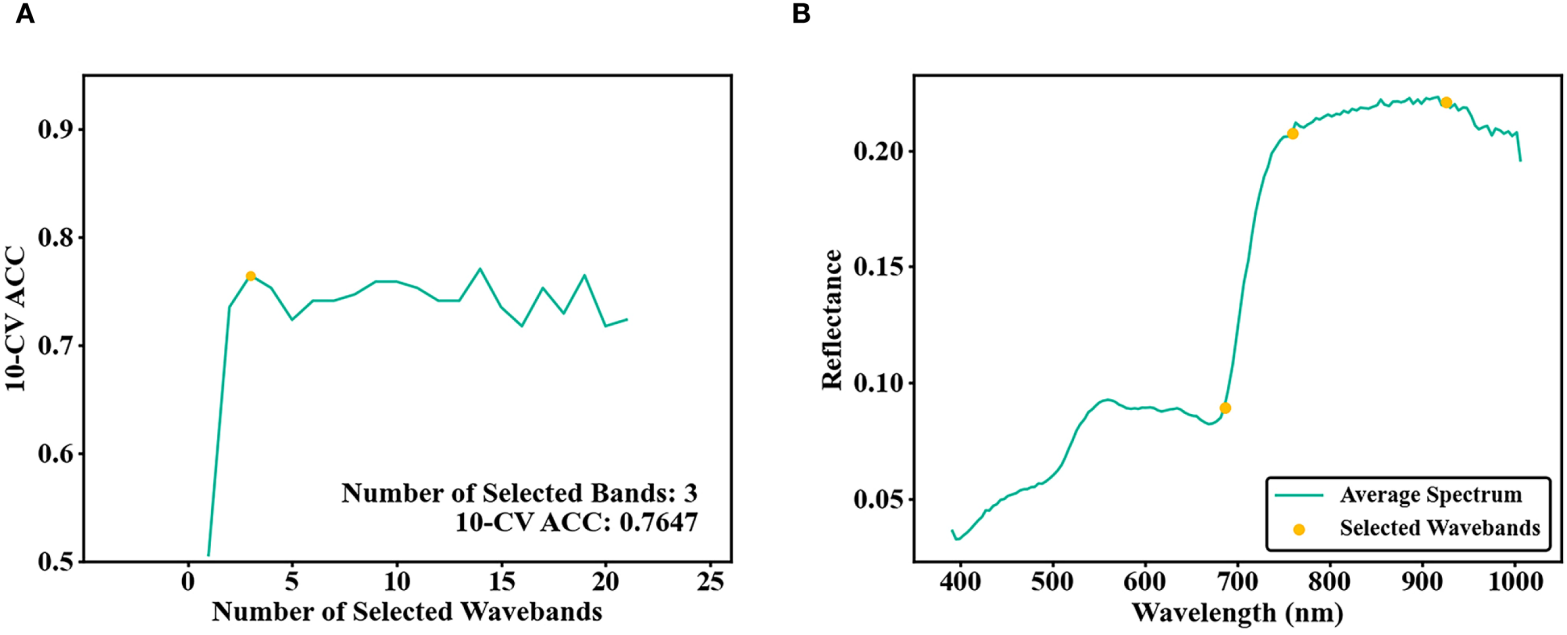
**(A)** Number of bands selected by ISIC-SPA and 10-CV ACC of the training set; **(B)** ISIC-SPA selected bands.

After secondary HSI band screening using ISIC-SPA-RF. the 10-CV ACC reached 0.7647, further increases in band numbers still caused minor fluctuations of 10-CV ACC around 0.76, with the overall trend stabilizing (**Figure 8A**). Based on the ISIC-SPA-RF results, 1 NIR bands 926 nm, 2 red band 686 nm and 759 nm were selected (**Figure 8B**).

### 3.2 Evaluation of band selection algorithms and screening of vegetation indices

The SPA-RF, ISIC-RF, and ISIC-SPA-RF methods were applied for band selection. The classification model was then constructed using RF based on the selected bands, and its performance was evaluated with test set accuracy metrics (**Table 2**).

**Table 2:** Test set performance of RF models constructed with different band selection algorithms.

Arithmetic	N	OA	Kappa	PA_mean_	UA_mean_
SPA	10	71.05%	0.57	72.59%	70.58%
ISIC	21	75.44%	0.63	76.34%	75.45%
ISIC-SPA	3	70.18%	0.55	71.03%	70.52%

The RF model based on the ISIC method showed the best performance. It had the highest OA, Kappa, UAmean, and PAmean. OA reached 75.44%, and Kappa was 0.63. The SPA method performed second best. OA and Kappa were 71.05% and 0.57 respectively. The ISIC-SPA method achieved only 70.18% OA. However, it used only 3 bands. This was much fewer than the 21 bands for ISIC and 10 bands for SPA. Therefore, considering both accuracy and band number, the 3 band ISIC-SPA method was selected for further vegetation index construction.

Four VIS were constructed based on the spectral bands selected through ISIC-SPA-RF. Subsequent correlation analysis evaluated relationships between these indices and tree damage progression stages. The four VIS with the highest Spearman correlation coefficients were selected: NDSI (759, 686), DSI (759, 686), RSI (759, 686), and RA (686, 759, 926).

### 3.3 Separability analysis of LiDAR metrics and VIs

**Figure 9:**
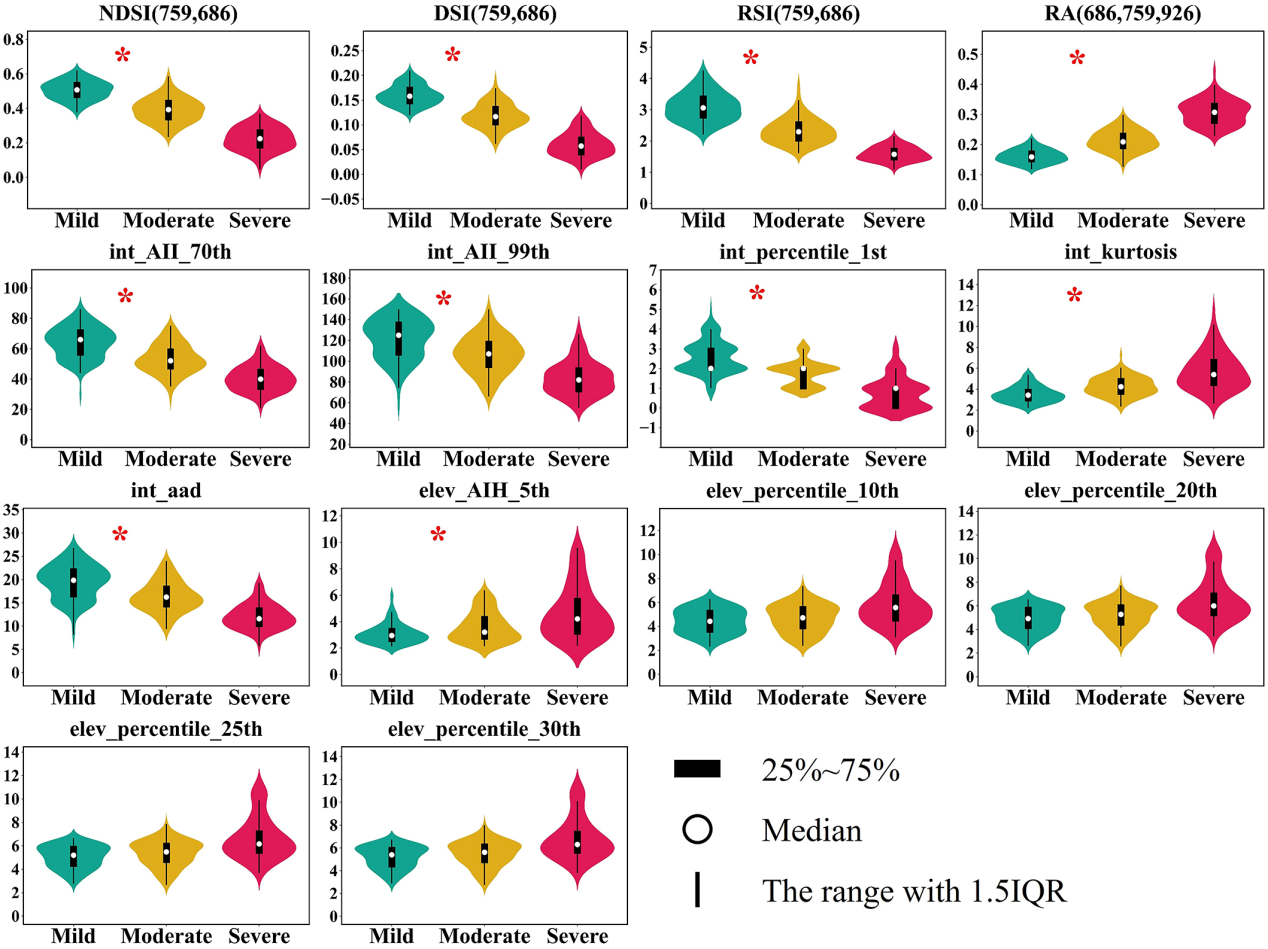
Vegetation indices and LiDAR metrics across three stages of damage. The symbol * indicates significant differences between mild and moderate stages.

The separability of four VIs and ten LiDAR metrics were shown in **Figure 9**. The K-W analysis indicates significant differences (P < 0.01) among the three damage stages for all 14 variables. Between the early and moderate damage stages, ten variables show overlap at the 25th and 75th percentiles. For the severe damage stage, five variables overlap at the 25th and 75th percentiles with the other two stages. Eleven variables differ significantly (P < 0.01) between the early and moderate damage stages. However, no significant differences are observed for elev_percentile_10th, elev_percentile_20th, elev_percentile_25th, and elev_percentile_30th, which may hinder the detection of early damage stages. Variables with significant differences (P < 0.01) were used as single or combined datasets to build RF classification models.

### 3.4 Classification results for different data sources

**Figure 10:**
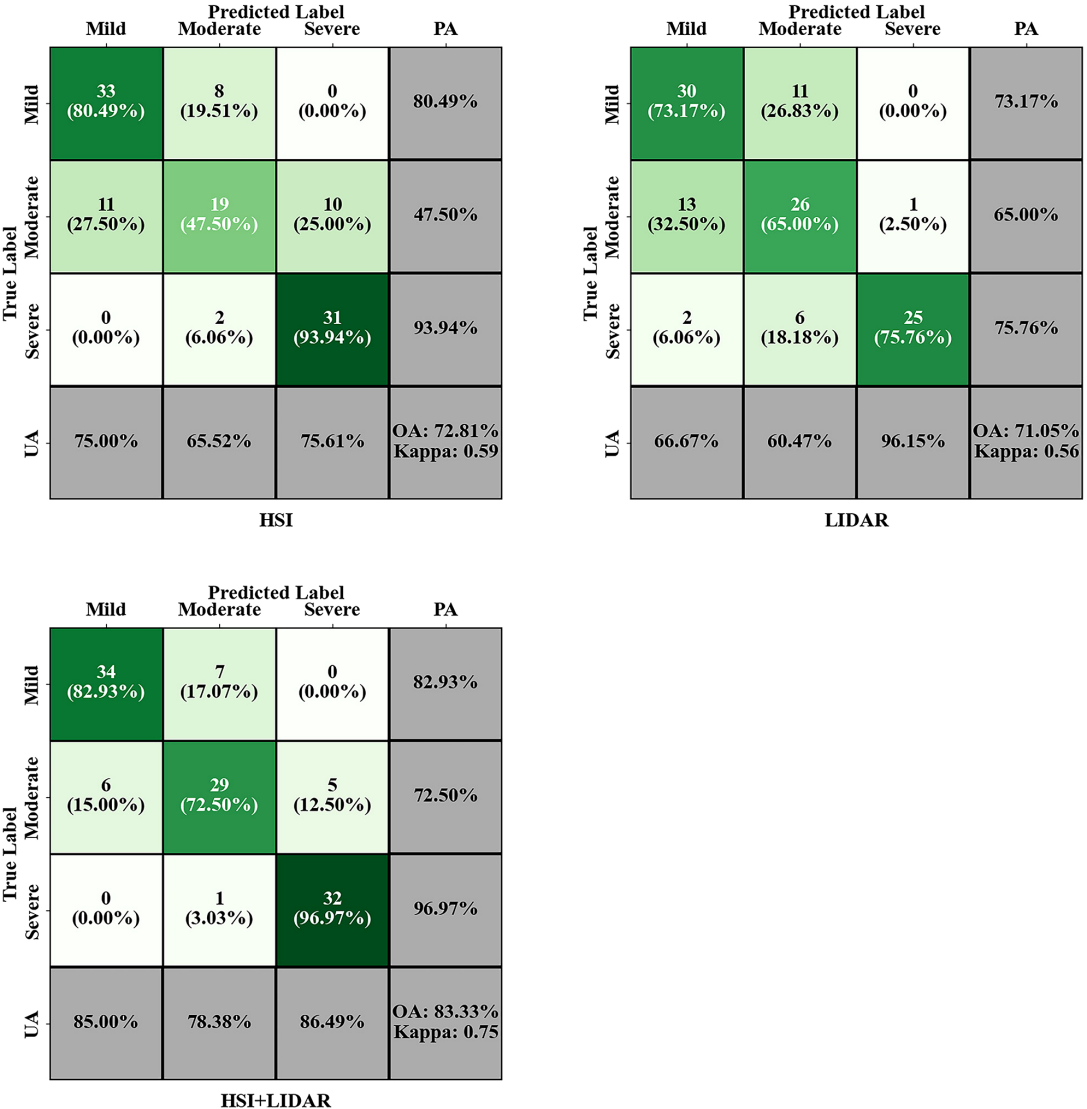
Confusion matrix results of RF classification.

The confusion matrix displayed classification results using VIs and LiDAR metrics as input data (**Figure 10**). The RF model using VIs as input data achieved an OA of 72.81% and a Kappa of 0.59. The severe stage showed the best classification performance, with an accuracy of 93.94%. followed by the mild stage, with an accuracy of 80.49%. The moderate stage achieved an accuracy of only 47.50%. The RF model constructed using LiDAR metrics achieved an OA of 71.05% and a Kappa of 0.56. Among the mild, moderate, and severe stages, the severe stage achieved the highest accuracy of 75.76%. The moderate stage showed the lowest accuracy of 65.00%.

The classification model built with the combined dataset of HSI bands and LiDAR metrics achieved an OA of 83.33% and a Kappa of 0.75, with the confusion matrix shown in **Figure 10**. The severe stage showed the best classification performance with an accuracy of 96.97%. The mild stage achieved an accuracy of 82.93% with eight samples misclassified as moderate. The moderate stage exhibited the poorest classification results. Compared to using VIs alone, this method increased OA by 10.52%, improved accuracy for mild and severe stages by 2.44% and 3.03% respectively, and raised accuracy for the moderate stage by 25%.

**Figure 11:**
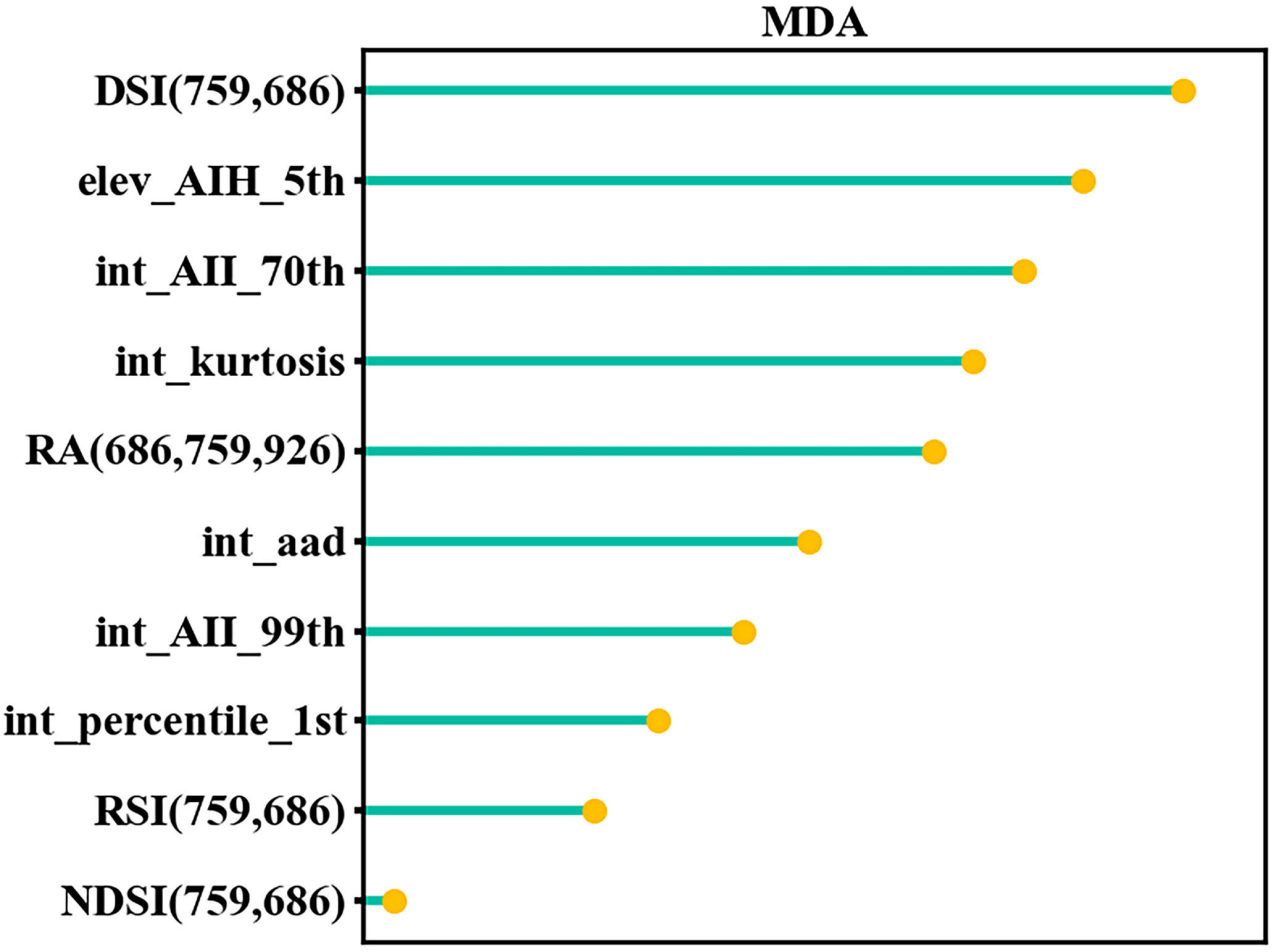
Variable importance ranking based on MDA.

The importance ranking of selected features using MDA in the RF model is shown in **Figure 11**. DSI (759, 686) was the most important HSI variable. Among LiDAR metrics, the elev_AII_5th is the most significant.

## 4 Discussion

This study used LiDAR and HSI data, combined with the bioecological characteristics of *Dendrolimus* spp. damage. Based on the defoliation percentage of *P. tabuliformis*, trees were classified into three damage stages: mild, moderate, and severe. Focusing on early stage damage caused by newly hatched or overwintering larvae, monitoring was conducted at the single tree level. The results showed that integrating LiDAR and HSI data improved the early detection accuracy of *Dendrolimus* spp. compared to single datasets, achieving effective results in identifying damaged trees.

### 4.1 Optimal band selection for hyperspectral

Among the bands select algorithms, the ISIC-SPA-RF method selected 3 bands, far fewer than the 10 bands of SPA-RF and the 21 bands of ISIC-RF, and achieved the best classification performance on the test dataset.

The ISIC-SPA-RF method selected four optimal bands at 686 nm, 759 nm and 926 nm. The 686 nm and 759 nm bands are red-edge bands linked to chlorophyll content and leaf area index (LAI) (Gholizadeh et al., 2016; Tao et al., 2020). HSI data were collected in early June during the peak feeding period of *Dendrolimus* spp., a period characterized by severe needle loss and reduced chlorophyll content. As defoliation increased, a blue shift in the red-edge bands occurred, resulting in higher reflectance. Meanwhile, severe needle loss reduced tree transpiration and water content (Li et al., 1997), resulting in lower reflectance in the NIR bands at 926 nm. When HSI and LiDAR data are combined, DSI (759, 686) was the most important HSI variable, matched the biophysical processes of *Dendrolimus* spp. damage in trees, and clearly showed differences between damage stages. The bands after dimensionality reduction and the constructed VIs achieved high accuracy. However, in practical applications, HSI data acquisition is costly, with large data volume and complex image processing. UAV based multispectral data (MSI) can replace HSI for band data collection. MSI and HSI not only show strong band compatibility but also reduce costs, improve processing efficiency, and facilitate large scale forest monitoring.

### 4.2 Integration of HSI and LiDAR data

HSI data effectively monitor *Dendrolimus* spp. damage in trees. Previous studies by Zhang et al. used HSI data and partial least squares regression (PLSR) to model needle loss in *P. tabuliformis*, achieving an R² of 0.8061 for mild to moderate damage stages (DP ≤ 40%) (Zhang et al., 2018). Bai et al. used spectral indices with multiple regression to quantify DP, yielding an R² value of 0.7860 (Bai et al., 2016). In comparison, the RF classification model developed in this study using hyperspectral data achieved an OA of 72.81%, with 80.49% accuracy for mild damage stages. The improved monitoring performance at early stages may be due to three reasons. First, compared to prior research, the classification standard was based on the effect of DP on volume growth in *P. tabuliformis*. At 30% DP, no significant growth reduction occurred; instead, enhanced tree height, apical shoot growth, and lateral shoot growth may have been detected. At 50% DP, a highly significant volume growth decrease was recorded. Using these thresholds, damage stages of *P. tabuliformis* were classified. Greater differences in DP between damage stages were present compared to conventional standards. Zhang et al. used only 12 mildly damaged samples, whereas 103 such samples were analyzed in this study, with regression analysis replaced by RF classification. These differences in sample size and methodology may explain the result variations.

The confusion matrix results show that LiDAR data can detect trees damaged by *Dendrolimus* spp., with an OA of 71.05%. In comparison, He-Ya et al. achieved 62% OA using LiDAR data and support vector machine (SVM) classification in a study on *Dendrolimus superans* (He-Ya et al., 2024). This difference may arise on one hand from variations in LiDAR point cloud density. The point cloud density during our data collection was 189 points/m², while that of previous studies was 70 points/m². Additionally, tree canopy occlusion reduces lower canopy point cloud density (Hamraz et al., 2017), leading to incomplete 3D tree models and less accurate structural parameter extraction (Cateanu and Ciubotaru, 2021). On the other hand, He-Ya et al. used only LiDAR height metrics, not intensity or density metrics. Our study focused on *P. tabuliformis* as the host species, whereas their study focused on larch. These differences in host tree species may have affected the classification results. LiDAR is less affected by environmental factors and has low requirements for illumination conditions. Additionally, the typically short peak feeding phase of *Dendrolimus* spp. and its rapid spread necessitate monitoring of large forest areas. LiDAR is less affected by environmental factors, allowing data acquisition even on cloudy days while covering wide areas during collection. When HSI or MSI are restricted by weather conditions, monitoring requirements can be met by using LiDAR alone.

Combining HSI and LiDAR data improved the classification accuracy of damaged trees, achieving an OA of 83.33%. This represents an 10.52% OA improvement compared to using VIs alone. The recognition accuracy for three damage stages increased by 2.44%, 25%, and 3.03%, respectively, with the mild damage stage reaching 82.93%. Although the classification performance for the moderate damage stage showed the greatest improvement, its accuracy remained the lowest, not exceeding 80%. Firstly, the moderate stage is a transitional phase between the mild stage and severe stage, with a highly variable mixture ratio of healthy to damaged needles. Trees with a DP close to 30% exhibit spectral and structural characteristics similar to the mild stage, while those with a DP near 50% are more similar to the severe stage, and lack typical and uniform characteristics unlike the other two stages. Various variables may exhibit greater variability within the group. Secondly, two-dimensional HSI imagery only monitors the upper part of the canopy, while damage from *Dendrolimus* spp. generally begins in the lower part of trees. This may cause changes in canopy spectral features during the moderate damage stage to lag behind the actual damage condition. Additionally, misclassification of damage stages due to human error during DP assessment further increases the difficulty of accurate classification.

The integration of the two datasets demonstrates complementary advantages. HSI band-combined VIs reduced background interference (Qiao et al., 2020), enhance vegetation information in spectral data, and effectively monitor spectral changes caused by physiological variations such as leaf yellowing, chlorophyll reduction, and water stress. LiDAR quantifies structural information linked to the damage characteristics of *Dendrolimus* spp. The elev_AIH_5th metric in LiDAR describes structural features of the lower tree layer. This may link to *Dendrolimus* spp. overwintering on the leeward side of trees, nearby stones, and leaf litter. These insects climb trees the next year and first affect lower needles. Additionally, damaged trees showed reduced water content and lower NIR reflectance. This decreased LiDAR point cloud intensity, causing intensity metrics to decline with higher defoliation rates. The int_kurtosis metric reflects heterogeneity in point cloud intensity distribution. This occurs because *Dendrolimus* spp. feeding reduces needles and increases exposed trunks. When weather allows, forest areas are small, or higher accuracy is needed, combining HSI and LiDAR allows physiological and structural data to complement each other, providing more accurate and reliable damage data.

### 4.3 Shortcomings and improvements

The integration of hyperspectral and LiDAR data enhanced the early monitoring accuracy for *Dendrolimus* spp., enabling reliable detection of trees at mild damage stages, though further optimization is warranted. During hyperspectral data collection, manual selection of tree crown ROIs may have introduced errors. Future studies should incorporate canopy segmentation algorithms to eliminate such subjective bias and enable efficient spectral data extraction from larger forest areas. This study was limited by its sample size and geographical scope. The restricted scale may affect the model’s generalizability across diverse geographical environments, forest stand structures, and site conditions. Subsequent research should establish sample plots across broader regions to validate and optimize the classification model. Furthermore, data collection was limited to a relatively short time window due to constraints in flight equipment and local pest control operations. Future work will explore long-term time-series monitoring using MSI and RGB as primary data sources to reveal the spatiotemporal dynamics of *Dendrolimus* spp. damage. We will also further investigate the relationship between LiDAR data and the biophysical processes of *Dendrolimus* spp. damage, and integrate LiDAR with MSI or RGB data to improve early detection accuracy and coverage in forest areas.

## 5 Conclusions

Effective forest pest management supports sustainable forest development. The combination of HSI and LiDAR technologies provided a novel approach for early monitoring of *Dendrolimus* spp. infestations. This study used HSI data to capture spectral features of damaged trees and LiDAR data to obtain structural features. red and NIR bands were more sensitive to *Dendrolimus* spp. induced tree damage than other bands and were more effective for vegetation index development. Using LiDAR data alone achieved an OA of 71.05% in distinguishing damaged trees, confirming its capability to monitor *Dendrolimus* spp. damage. Combined HSI and LiDAR data improved monitoring results. The accuracy in detecting trees at mild damage stages increased by 2.44% and 9.76% compared to using VIS or LiDAR metrics alone, with an OA of 83.33%. The integration of HSI and LiDAR enhanced monitoring precision at the individual tree stage, offering a vital technical solution to prevent *Dendrolimus* spp. outbreaks.

## Data Availability

The datasets presented in this study are not readily available because they are part of an ongoing research project. Requests for data access should be directed to the authors. Requests to access the datasets should be directed to Rui Tang trtr955@bjfu.edu.cn.
